# Prospects and Challenges of the Drug Delivery Systems in Endometriosis Pain Management: Experimental and Theoretical Aspects

**DOI:** 10.1155/2021/2727174

**Published:** 2021-12-15

**Authors:** Bogdan Florin Toma, Razvan Socolov, Ovidiu Popa, Demetra Socolov, Irina Nica, Maricel Agop, Decebal Vasincu, Mihaela Grigore, Lacramioara Ochiuz

**Affiliations:** ^1^“Grigore T. Popa” University of Medicine and Pharmacy, Iasi 700115, Romania; ^2^Department of Obstetrics and Gynecology, “Grigore T. Popa” University of Medicine and Pharmacy, Iasi 700115, Romania; ^3^Department of Emergency Medicine, “Grigore T. Popa” University of Medicine and Pharmacy, Iasi 700115, Romania; ^4^Department of Odontology, Periodontics and Fixed Restoration, “Grigore T. Popa” University of Medicine and Pharmacy, Iasi 700115, Romania; ^5^Department of Physics, “GheorgheAsachi” Technical University of Iasi, Iasi 700050, Romania; ^6^Romanian Scientists Academy, Bucharest 050094, Romania; ^7^Department of Dental and Oro-Maxillo-Facial Surgery, “Grigore T. Popa” University of Medicine and Pharmacy, Iasi 700115, Romania; ^8^Department of Pharmaceutical and Biotechnological Drug Industry, “Grigore T. Popa” University of Medicine and Pharmacy, Iasi 700115, Romania

## Abstract

Endometriosis is considered a serious public health issue because of the large number of females affected by this illness. Chronic pain management in patients with endometriosis demands new strategies to increase the life quality of these patients. The development of drug delivery systems represents a new approach in pain treatment among endometriosis patients. Diclofenac sodium, one of the most utilized nonsteroidal anti-inflammatory drugs (NSAID), has its own limitations when being used in formulas such as oral, parental, or local applications. In this paper, a series of four drug release formulations based on chitosan, 2-hydroxy-5-nitrobenzaldehyde, and diclofenac sodium salt were prepared in view of the investigation of the drug release ability. The formulations were analyzed from a morphological and supramolecular point of view by scanning electron microscopy and polarized light microscopy. The in vitro drug release ability was investigated by mimicking a physiologic environment. A mathematical model, using the fractal paradigm of motion, is utilized to explain the behaviors of the drug delivery system presented in this paper. These results suggest a great potential of the proposed drug delivery system, based on chitosan and 2-hydroxy-5-nitrobenzaldehyde to improve the diclofenac sodium salt bioavailability, and it may represent a future treatment formula for endometriosis pain.

## 1. Introduction

Endometriosis is a wide benign, chronic, and inflammatory pathology among fertile women that is characterized by painful symptomatology and infertility. The distinctive mark of endometriosis diagnosis is represented by the presence of stromal and glandular endometrial tissues outside the uterus. The symptoms related endometriosis comprises dysmenorrhea, dyspareunia, and pelvic or lower abdominal pain that frequently has a negative impact on the patient's life quality, career, daily activities, relationships, and fertility. Sometimes, patients may accuse cyclical pain in other areas correlated with endometriosis [1]. Even if endometriosis is a very popular condition, the diagnosis can be difficult, especially in the less severe stages (stages I-II), and at this moment, laparoscopy is considered the “gold standard” for diagnosis [2].

The mechanisms of endometriosis are not entirely understood. It is believed to be an inflammatory condition that involves various endocrine, genetic, immunological, and environmental interplays with great impact in the initiation and progression of the pathology. The disfunction of the immunological system plays a critical role for the development and persistence of endometrial implants inside the peritoneal cavity. Peritoneal fluid represents an important immunological barrier system that contains different immune cells such as mesothelial cells, macrophages, natural killer (NK) cells, T and B lymphocytes, and monocytes. Immunoinflammatory factors, angiogenic factors, and endocrine pathways establish specific and dynamic circumstances that are necessary to create and grow endometriotic implants. The macrophage population is higher within peritoneal fluid and endometriotic implants and contributes to the inflammatory environment but, compared with nonendometriozic patients, presents a decreased phagocytic function and low expression of B scavenger receptor CD36. The ratio between M2 anti-inflammatory macrophages and M1 proinflammatory is inverted in endometriosis patients. An increase level of M1 macrophages found in endometriosis tissue contributes to profibrotic activity, survival, and progression of ectopic implants by angiogenesis and immune tolerance induction [3, 4]. Moreover, oestrogen receptors (ER) may play an important role in macrophage regulation, suggesting a correlation between immunological response and oestrogens. In endometriosis patients, it was shown that ER-*α* expression is positively linked to proinflammatory cytokine expression in macrophages and ER-*β* presents anti-inflammatory function [5]. An increased number of proinflammatory cytokines were found within endometrial implants. Therefore, ectopic implants showed a higher expression of transcription factor, nuclear factor-kB (NF-kB), along with fibronectin, intercellular adhesion molecule 1 (ICAM1), insulin-like growth factor I (IGFI), tumor necrosis factor-*α* (TNF-*α*), interleukin-6 (IL-6), and interleukin-8 (IL-8) which enhances growth function within the ectopic implant by promoting the proinflammatory environment [3]. These cytokines launch and enhance the inflammatory response, targeting the recruitment of various proinflammatory cells and mediators. Tumor necrosis factor and its receptors, TNFR1 and TNFR2, represent an extrinsic apoptosis pathway involved in endometriosis genesis, being implicated in inflammatory and endometrial repair [6, 7].

Natural killer (NK) cells are normally abolished by the peritoneal barrier environment, but within endometriosis patients, an overexpression of different surface receptors that can activate or suppress their function was found. They represent cytotoxic effector lymphocytes that do not need a major histocompatibility complex or previous exposure to the antigen to lyse the target cells. Lately, research has been focused to identify various factors, which may suppress NK cell cytolytic function such as IL-6, IL-15, and TGF-*β*1 [8–11]. The endocrine premature dendritic cells reach maturity and are carried through the lymphatic vessels in response to foreign antigens or various antigens on top of T cells from inflammatory targets. In endometriozic tissues, this physiological process is being modified and the population of CD83+ dendritic cells is significantly decreased, leading to endometrial antigen misrecognition by the circulating antiendometrial stromal cells [7].

Inflammation represents an important key in endometriosis pathogenesis, and further studies focused on the intracellular signaling mechanisms will contribute to understand better the inflammatory pathogenesis of endometriosis to develop future therapeutic strategies.

The treatment of symptoms is very wide, having various options, but the underlying pathology frequently demands repeated medical and surgical interventions. The possibilities of medical treatment include oral contraceptives, testosterone derivatives, progestogens, and gonadotropin-releasing hormone (GnRH) agonists. Regarding the surgical approach, there are two modalities used for endometriosis treatment such as ablative techniques and excision [12, 13]. In the management of pain-related endometriosis, they are utilized as first-line therapy nonsteroidal anti-inflammatory drugs (NSAIDs) which represent a group of analgesic drugs. This drug class inhibits the cyclooxygenase- (COX-) 1 and COX-2 enzymes. The COX-2 enzyme is responsible for prostaglandin formation, an important key in inflammatory response initiation, and its inhibition determines therapeutic anti-inflammatory effects. Diclofenac sodium is a traditional NSAID that inhibits both COX-1 and COX-2 with greater impact on COX-2, being comparable to celecoxib, a first generation of the COX-2 inhibitor [14]. Depending on the dose that is used and the time between administrations, diclofenac like other COX-1 and COX-2 inhibitors, associates an increased risk of gastrointestinal, cardiovascular, and renal complications. To reduce the side effects and to improve the variability of diclofenac indications, the pharmaceutical industry developed different formulas with large approaches such as oral, parental, and local applications.

Drug delivery is a research direction of high contemporary interest, meant to improve the bioavailability of therapeutic drugs, to overcome impairments such as limited drug solubility or tendency of aggregation and to limit their side effects by targeted delivery. In time, many types of drug carriers were proposed to fulfill the requirements of in vivo drug release, such as liposomes, hydrogels, nanogels, and micelles [15–18]. Among them, the hydrogels present the advantage of high similarity with human tissues, while those based on natural or derivatives of natural have good biocompatibility and biodegradability. Along this line of thought, chitosan-based hydrogels proved the potential to skip the barrier towards real-world applications, because besides biocompatibility and biodegradation, it has also a large realm of biologic properties [19]. Recent research in the area of chitosan hydrogels revealed a new crosslinking method with monoaldehydes, based on a combined physicochemical method consisting in the self-assembling of the newly formed imine units into ordered clusters which play the role of crosslinking nodes [20–23]. This nontraditional hydrogelation method proved a great potential for the design of drug delivery formulations, bringing the advantage of the use of biocompatible natural aldehydes with synergic biologic properties [24–26]. In this context, hydrogels prepared from chitosan and a vanillin derivative, 2-hydroxy-5-nitrobenzaldehyde, showed thixotropic behavior [24] and antimicrobial activity [27], promising to be an excellent matrix for the local delivery of diclofenac for the treatment of endometriosis. To further understand the mechanism behind the slow release of drugs from these chitosan-based hydrogels, a multifractal mathematic model is proposed to explain the drug delivery complex mechanisms.

## 2. Materials and Methods

### 2.1. Materials

Chitosan of low molecular weight (217.74 kDa, DA: 85%), 2-hydroxy-5-nitrobenzaldehyde (98%), diclofenac sodium salt (DCF) (99%), and phosphate buffer solution from Aldrich were used as received.

### 2.2. Preparation of the Formulations

A series of four drug delivery formulations were prepared by in situ crosslinking of chitosan with 2-hydroxy-5-nitrobenzaldehyde in the presence of diclofenac sodium salt, according to reference [16]. Shortly, (i) a chitosan solution was prepared by dissolving it in 0.7% acetic acid to give a 2% solution, (ii) a 1% solution of 2-hydroxy-5-nitrobenzaldehyde in ethanol was mixed with DCF, and then, (iii) it was slowly poured into the chitosan solution under vigorous magnetic stirring. The quantities of chitosan and 2-hydroxy-5-nitrobenzaldehyde were calculated to reach four different crosslinking degrees in the final formulations, corresponding to four different ratios of the amine and aldehyde functional groups: 5/1, 4/1, 3/1, and 2/1. The diclofenac amount was kept constant, consistent with the accepted dose (g/kg). The formulation codes were formed from the number corresponding to the ratio of functional groups and the letter D of DCF: 5D, 4D, 3D, and 2D.

### 2.3. Methods and Equipment

The formulations were frozen in liquid nitrogen and then lyophilized using a LabconcoFreeZone Freeze Dry System equipment for 24 h at −54°C and 1.512 mbar, to obtain the corresponding solid state as xerogels.

The morphology of the formulations was investigated on the corresponding xerogels, using a field emission scanning electron microscope (SEM) EDAX—Quanta 200 at an accelerated electron energy of 20 KeV.

The supramolecular architecture of the formulations was observed by polarized light microscopy (POM) with a Leica DM 2500 microscope, on slim slices of xerogels placed between two lamellae.

*In vitro* investigation of the DCF release from formulations was investigated applying a standard procedure [28]. Briefly, the formulation samples were immersed into vials containing 10 mL of phosphate buffer and maintained at 37°C. At certain moments, 2 mL of the supernatant was withdrawn and replenished with fresh buffer solution. The concentration of DCF released into the supernatant was assessed by measuring the specific DCF absorbance and its fitting to a calibration curve. The experiments were performed in triplicate. The absorbance spectroscopy was done on a HORIBA spectrophotometer.

## 3. Results and Discussions

A series of four formulations were prepared by chitosan hydrogelation with 2-hydroxy-5-nitrobenzaldehyde in the presence of diclofenac sodium salt. The designing of these formulations considered the properties of the components and the intermolecular forces which can be developed between them. Thus, chitosan is a well-known biopolymer with excellent biocompatibility and biodegradability and valuable biologic properties such as antimicrobial activity and blood clotting, hypocholesterolemic, or immunoenhancing effects. 2-Hydroxy-5-nitrobenzaldehyde has been chosen as a chitosan crosslinker, due to the fact that it is a vanillin derivative, nontoxic for the human body, and with good antimicrobial properties [27, 29], having promising synergistic effect with diclofenac drug. The chemical structure of the three components displays polar groups such as –Cl, –OH, –COO–, and –NO_2_, which promotes intermolecular forces among the three components (such as H-bonds and polar forces) creating the possibility of a prolonged release of the drug and thus a prolonged bioavailability. To appreciate the influence of the matrix on the release kinetic of the drug, four formulations were prepared by varying the ratio between the amine and aldehyde groups and consequently the crosslinking density.

**Figure 1:**
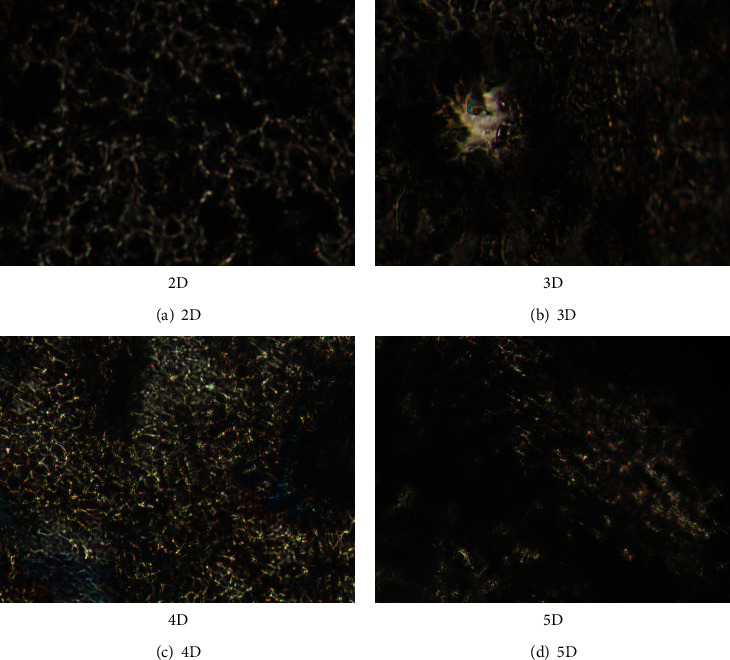
Birefringent textures of the 2D–5D formulations evidenced by POM.

Polarized light microscopy was used to assess the encapsulation of the drug into the matrix (Figure 1). The formulations revealed birefringent banded textures, signatures of the layered phases [20, 24, 30], confirming thus that the self-assembling of the imine units formed between chitosan and 2-hydroxy-5-nitrobenzaldehyde was the main promotor of formulation hydrogelation [20–24]. This texture pattern was evident for all four formulations, signifying that the DCF presence did not hamper the hydrogelation for any of them. Besides, the texture was continuous, without crystals, suggesting that DCF molecules were dispersed into the hydrogel matrix at least at the submicrometric level, under the evaluation limits of the POM [28].

**Figure 2:**
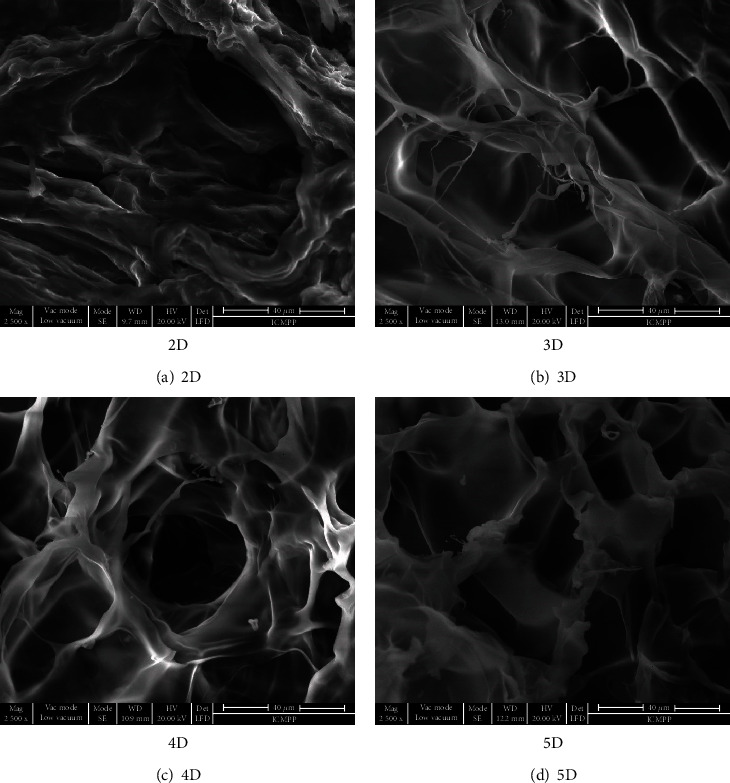
Microstructure of the studied formulations visualized by SEM.

As the formulation morphology is in an important factor affecting the drug kinetics release, scanning electron microscopy was performed to have a better understanding of it.  Figure 2 shows that the microstructure of the formulations was not significantly affected by the crosslinking degree. Except for the 2D formulation, which showed a more compressed structure with no clear pores, the other samples revealed a porous morphology, with well-delimited interconnected pores with a diameter around 50 *μ*m. Compared to the neat hydrogels without drug, their pore walls were thick, indicating the encapsulation of DCF into them [24]. This hypothesis is supported by the strong interactions which can develop between the DCF and the hydrogel matrix, which clearly prompted the drug anchoring into matrix. In this view, it can be expected that the diffusion of the DCF molecules through the matrix will be retarded, promoting its prolonged release [31].

**Figure 3:**
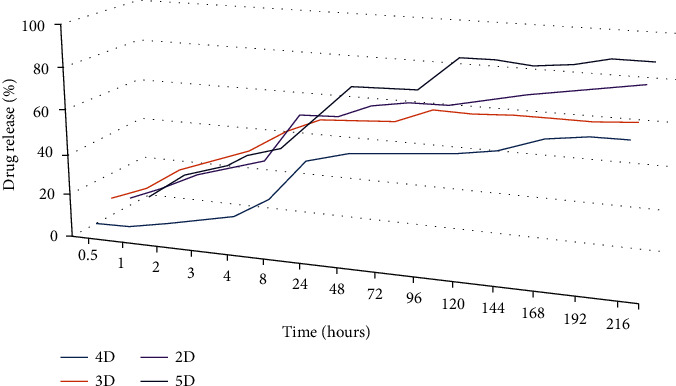
Drug release profile of DCF from formulations and the corresponding exponential trend line.

The *in vitro* release of DCF from the formulations was monitored by applying conditions which mimic the physiologic environment. As can be seen in Figure 3, the DCF was released in a pulsatile manner, no matter what was the crosslinking degree of the matrix [32]. Taking into consideration the influence of the drug size on the dissolution rate, this behavior can be correlated with the encapsulation of the DCF into the formulations as submicrometric crystals of different sizes [24]. Furthermore, no clear correlation of the release profile to the crosslinking degree was distinguished. The hydrogel matrix with the lowest crosslinking degree (5D) was favorable towards a fast release of almost all DCF amounts over 9 days. On the contrary, the formulation with the highest crosslinking degree (2D) presented a more rapid release compared to those with a medium crosslinking degree (3D and 4D), attaining more than 80% DCF release compared to less than 70%. Nevertheless, the exponential trend line showed a continuous release of the drug for the entire investigation period (Figure 3). This release behavior, which did not match to a clear rule, has been correlated with the dissimilar viscosity of the hydrogelation system, influencing the DCF crystallization, i.e., the growing of crystals of different sizes.

## 4. Theoretical Model

Taking into account the complexity of the phenomena that occur in release processes (drug diffusion, erosion of polymer matrix, drug solubility, etc.), it is admitted (evidently, as a work hypothesis) that this “complexity” can be “covered” by multifractality. In other words, the polymer-drug complex system release dynamics will be described through continuous and nondifferential curves (multifractal curves and not monofractal curves, i.e., of a single fractal dimension *D*_*F*_, as is the usual case in [33]). Then, the multifractal theory of motion in its hydrodynamic form becomes functional through the following equations [34, 35]:

(1) $$\begin{array}{c} \partial_{t}V_{D}^{i}+V^{l}\partial_{l}V_{D}^{i}=-\partial^{i}Q, \end{array}$$

(2) $$\begin{array}{c} \partial_{t}\rho +\partial^{l}\left(\rho V_{D}^{l}\right)=0, \end{array}$$

(3) $$\begin{array}{c} Q=2\lambda^{2}{\left(dt\right)}^{\left[2/f\left(\alpha \right)\right]-1}\frac{\partial_{l}\partial^{l}\sqrt{\rho }}{\sqrt{\rho }}, \end{array}$$

(4) $$\begin{array}{c} \partial_{t}=\frac{\partial }{\partial t},\partial_{l}=\frac{\partial }{\partial X^{l}},\partial_{l}\partial^{l}=\frac{\partial }{\partial X^{l}}\left(\frac{\partial }{\partial X^{l}}\right),\;i,l=1,2,3. \end{array}$$

In relations (1)–(4), the terms have the following meanings:

- *t* is the nonfractal time having the role of an affine parameter of the release curves
- *X*^*l*^ is the multifractal spatial coordinate
- *V*_*D*_^*i*^ is the “multifractal fluid” velocity on a differentiable scale resolution (the polymer-drug complex system is assimilated to a “multifractal fluid”; for details on the “behavior” of such a “physical object,” see [33–35])
- *ρ* is the state density of the “multifractal fluid”
- *λ* is the structural constant specific to the release process associated to the multifractal—nonmultifractal transition
- *dt* is the scale resolution
- *f*(*α*) is the singularity spectrum of order *α* dependent on the fractal dimension *D*_*F*_ [36, 37]

Operating with multifractal “manifolds” instead of monofractal ones (in the case of dynamic release systems) has some advantages:

- Areas of the polymer-drug complex system of a certain fractal dimension may be identified and can be characterized from a release dynamic viewpoint. From here, the number of zones of the polymer-drug complex system which have their fractal dimension in a certain interval of values may be identified
- Universality classes can be identified in the domain of dynamic release systems, even when the attractors have different aspects

Equation (1) corresponds to the multifractal law of specific momentum conservation and equation (2) corresponds to the multifractal conservation law of state density, while equation (3) corresponds to the multifractal specific scalar potential as a measure of the multifractalization degree of the release curves.

Introducing the fractal state function of the form

(5) $$\begin{array}{c} \psi =\sqrt{\rho }\;\exp\left(\text{is}\right),\;i=\sqrt{-1}, \end{array}$$

where $\sqrt{\rho }$ is an amplitude and *s* is a phase, then, two types of velocities can be defined:

- *V*_*D*_^*i*^ velocity at differentiable scale resolution

(6) $$\begin{array}{c} V_{D}^{i}=2\lambda {\left(dt\right)}^{\left[2/f\left(\alpha \right)\right]-1}\partial^{i}s \end{array}$$

(ii) *V*_*F*_^*i*^ velocity at nondifferentiable scale resolution

(7) $$\begin{array}{c} V_{F}^{i}={\left(dt\right)}^{\left[2/f\left(\alpha \right)\right]-1}\partial^{i}\ln\rho \end{array}$$

Now, the synchronization of the dynamics at the two scale resolutions, equivalent to the controlled drug release process, implies the operation with the following constraint:

(8) $$\begin{array}{c} V_{D}^{i}=-V_{F}^{i}. \end{array}$$

In this condition, the multifractal conservation law of state density transforms into a diffusion equation of multifractal type:

(9) $$\begin{array}{c} \partial_{t}\rho =\lambda {\left(dt\right)}^{\left[2/f\left(\alpha \right)\right]-1}\partial_{l}\partial^{l}\rho =\sigma \partial_{l}\partial^{l}\rho . \end{array}$$

**Figure 4:**
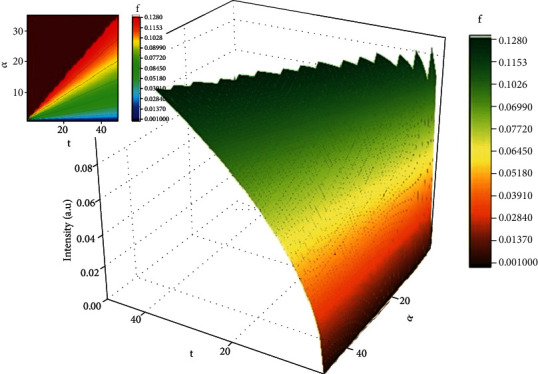
3D (left side) and contour plot (right side) representations of the multifractal function used for drug release mechanism analysis.

It results that these “mechanisms” “manifest”/are “perceived” as diffusions at various scale resolutions in a multifractal space (Fickian-type diffusion, non-Fickian-type diffusion, etc.). To explain this situation it should be considered the one-dimensional drug diffusion of multifractal type from a controlled-release polymeric system with the form of a plane shut, of thickness *δ*. If drug release of the multifractal type occurs under perfect sink condition, the following initial and boundary conditions can be assumed:

(10) $$\begin{array}{c} t=0,\\ -\frac{\alpha }{2}<x<\frac{\alpha }{2},\\ \rho =\rho_{0}\\ t>0,\\ x=\pm \frac{\alpha }{2},\\ \rho =\rho_{1}, \end{array}$$

where *ρ*_0_ is the initial drug state density of the multifractal type in the “device” of the multifractal type and *ρ*_1_ is the drug state density at the “polymer-fluid” interface of the multifractal type. This solution equation under these conditions can take the following form (for details in the classical case, see [38, 39]). In Figure 4 shows the multifractal function representation utilized to analyze the drug release

(11) $$\begin{array}{c} f=\frac{\rho_{t}}{\rho_{\infty }}=2{\left(\frac{\sigma t}{\delta^{2}}\right)}^{1/2}=\left\{\pi^{-1/2}+\overset{\infty }{\underset{n=1}{\sum }}{\left(-1\right)}^{n}\mathrm{erfc}\left[\frac{n\delta }{2{\left(\sigma t\right)}^{1/2}}\right]\right\}. \end{array}$$

An accurate expression can be obtained for small values of *t* since the second term of (11) disappears, and then, it becomes:

(12) $$\begin{array}{c} \frac{\rho_{t}}{\rho_{\infty }}=2{\left(\frac{\sigma t}{\delta^{2}}\right)}^{1/2}=\text{const}{\left(t\right)}^{1/2}. \end{array}$$

**Figure 5:**
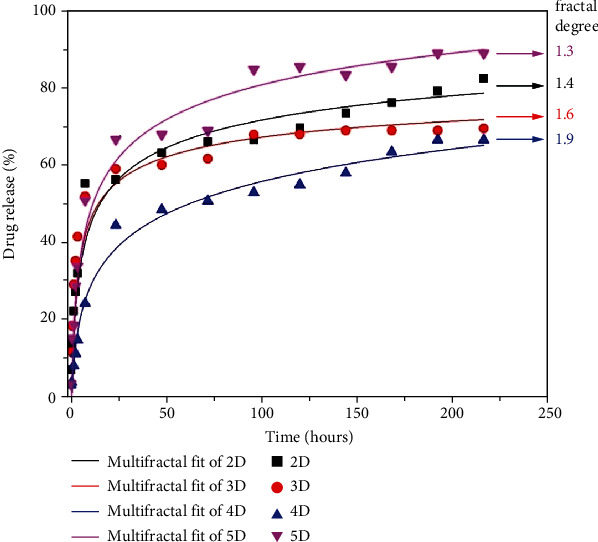
Experimental showcase of the DCF release from formulations fitted by the multifractal theoretical model.

In such a context, *ρ*_*t*_/*ρ*_∞_ can be assimilated to the fraction of dissolved drug, i.e., *M*_*t*_/*M*_∞_ ≡ *ρ*_*t*_/*ρ*_∞_, where *M*_*t*_ is the amount of drug dissolved in time *t* and *M*_∞_ is the total amount of time dissolved when the pharmaceutical dosage form is exhausted [40, 41]. The confirmation of the model is presented in Figure 5, for the release of DCF from the chitosan-based matrix. The empirical data was fitted with the multifractal function. The model is well equipped to predict the drug release dynamics [38]. The use of any classical model to fit the in vitro release will not offer any information regarding the mechanism of the drug release, as there are a wide span of factors influencing the release process. Concerning the theoretic model developed in the multifractal paradigm, this can be validated through an adequate calibration on the empirical data, by choosing the constants according to the particularities of our polymer-drug system followed by a normalization of the data. The calibration process is not a trivial one as it strictly depends on the nature of the phenomena investigated; the method was previously tested for other physical phenomena with promising results [42–63]. We can observe that the model fits well all data sets. The saturation is usually reached at around 24–28 hours depending on the formulation and its corresponding fractal degree. This is also due to the morphology of the formulation which has a more organized structure enhancing the release; thus, a link can be made between the differential parameters defining the morphology of the polymer and the fractal degree defining the collective movement of the drug release scenario in a multifractal model. When we further analyze these results in the fractal paradigm, it results that a nonfractal morphology will lead to a higher fractality of the release drug geodesics as it enhances the interactions between the drug and the release media. As the morphology of the polymer formulations becomes fractalized, the release is reduced and the overall fractalization degree of the drug release is reduced.

## 5. Conclusions

A series of four drug release formulations were prepared by in situ hydrogelation of chitosan by with 2-hydroxy-5-nitrobenzaldehyde in the presence of diclofenac sodium salt as a drug model. The POM and SEM measurements emerged to the conclusion that the formulations have a homogenous dispersion of the drug into the pore walls at the submicrometric level. The size of DCF crystals appeared to vary depending on the system viscosity during the hydrogelation. This favored a pulsatile prolonged release of the drug over 9 days. The mathematical model was performed in the framework of the scale relativity theory and validated by our analysis and experimental data.

Because pain is the most common unpleasant symptom associated with endometriosis or deep infiltrative endometriosis, the current research on NSAIDs and the development of drug delivery systems can open new future perspectives on management of this category of patients. Drug delivery systems already play an important role in reducing symptoms related endometriosis, showing great improvement in the management of this debilitating condition.

## Data Availability

The data used to support the findings of this study are included within the article.
